# Recovery of *Abies alba* and *Picea abies* saplings to browsing and frost damage depends on seed source

**DOI:** 10.1002/ece3.4955

**Published:** 2019-02-27

**Authors:** Andrea Doris Kupferschmid, Caroline Heiri

**Affiliations:** ^1^ Swiss Federal Research Institute WSL Birmensdorf Switzerland; ^2^ Amt für Wald des Kantons Bern Bern Switzerland

**Keywords:** climate change, common garden, evolutionary adaptation, herbivory, provenance test, tree regeneration, ungulate browsing

## Abstract

The density of wild ungulates has increased in the last century, and browsing has become a major driver of forest succession in the northern hemisphere. In addition, tree species are expected to respond differently to future climate conditions, especially an increased frequency of late frost events. The aim of this study was to analyze the influence of intraspecific genetic variation on the recovery of two tree species to frost and browsing. An experiment with saplings from 90 *Abies alba* and 72 *Picea abies* seed sources was conducted. Five‐year‐old saplings were clipped at three intensities before budburst in spring. Growth (height, diameter, leader shoot length, and biomass) and quality (e.g. stem form, multistemming, reaction type) were assessed before and 1–2 years after clipping or 3–4 years after natural frost events, and provenance differences were related to environmental differences at the seed source. For *Abies*, frost and clipping resulted in reduced height growth in the first year after the stress and reduced height for two (clipping) to four (frost) vegetation periods. Sapling biomass, diameter increment, and quality decreased after heavy clipping. For *Picea*, which grew twice as fast as *Abies*, such effects were only found after frost damage. Population differences were significant for both species for all investigated growth traits and for *Picea* also for some quality variables. The “reaction type” after browsing (e.g. new shoot, existing twig bending upward) seems to be species specific and independent of seed source. In contrast, the time lag between clipping and formation of a clear new leader shoot increased for *Abies*with lower temperatures at the seed source. Lowland populations with warmer climates grew faster, and for *Picea* also qualitatively better, and recovered faster from leader shoot loss (*Abies*) or reacted at the uppermost meristem *(Picea*). Thus, the investigated stressors increased the existing differences among populations.

## 1 INTRODUCTION

There is growing evidence of increasing temperatures and decreasing summer precipitation in Central Europe (IPCC, 2013). Climate change is likely to affect trees on many sites (Westerling, Hidalgo, Cayan, & Swetnam, 2006). Although the expected increase in the frequency of drought periods or late frost events is expected to affect individual tree species differently (Richter et al., 2012). However, the existence of intraspecies differences is also well known from provenance trials. Apart from differences in height growth, second flushing and phenology (Frank et al., 2017; Szeligowski, Bolibok, Buraczyk, & Drozdowski, 2011), considerable differences have been observed between populations in frost resistance (Hansen & Larsen, 2004) and drought tolerance (Csillery, Ovaskainen, Sperisen, Widmer, & Gugerli, 2018). For this reason, populations that do well under future climate conditions would be beneficial for forestry purposes.

However, tests should be completed to determine if ungulate browsing does not interfere with growth to such a degree that it counters the growth benefits of climatically better‐adapted populations. In the last century, the density of chamois, roe, and red deer has increased (Apollonio, Andersen, & Putman, 2010). Therefore, ungulate browsing has become a major driver of forest succession in the northern hemisphere and can challenge the establishment of future tree generations (Tanentzap et al., 2009; Tremblay, Huot, & Potvin, 2007). In the face of increasing forest regeneration problems due to these high ungulate densities (Ramireza, Jansenb, & Lourens, 2018), it would be valuable to have better knowledge on the following: (a) the selective browsing on certain populations and (b) the recovery potential of different tree populations following browsing.

It is well known that browsing is inter‐ and intraspecifically a very selective process. For example, vigorously growing saplings are preferentially selected by ungulates (Iason, Duncan, Hartley, & Staines, 1996; Kupferschmid, 2018). Population differences in bud break and growth cessation have been shown to cause large differences in moose browsing in Finland (Viherä‐Aarnio & Heikkilä, 2006). Clear differences in browsing frequency have also been found between populations of *Eucalyptus morrisbyi* trees (Mann et al., 2012). Apart from traits that help a plant avoid browsing selection, those that enable a plant to recover can be seen as a strategy to mitigate browsing impacts (O'Reilly‐Wapstra et al., 2014).

Very few studies have been conducted to investigate the population differences in recovery following browsing. The growth trait differences in five populations of *Pinus sylvestris* were found to be maintained irrespective of browsing (O'Reilly‐Wapstra et al., 2014), and the same was found for saplings of 77 *Fagus sylvatica* seed sources (Frank, Heiri, & Kupferschmid, 2019). However, the compensation capacity of tree species in response to leader shoot browsing depends on many factors, among them the architecture of the tree saplings (cf. review by Kupferschmid, 2017). Therefore, predictions of no genetic differences in recovery from browsing for all tree species based on findings from *P. sylvestris* and *F. sylvatica* alone cannot be considered reliable.

In this study, we were interested in the population‐specific reaction to browsing and the variation in recovery from leader loss of two commercially very important tree species of Central Europe, that is, *Picea abies* (L.) Karst. and *Abies alba* Mill. We knew from the results of common garden experiments that *Picea* has considerable growth differences between populations and that *Abies* has at least some differences (Csillery et al., 2018; Frank et al., 2017). Further*,*saplings of *A. alba*are browsed much more frequently and heavily by ungulates than saplings of *P. abies* (Kupferschmid, 2018; Vacek et al., 2014), which could have led to different genetic selection and thus to differences in the genetic variation in the recovery following browsing. Additionally, recent browsing experiments have already led to recommendations to foresters that *A. alba* populations producing many buds should be planted at sites with high ungulate density based on the assumption that bud formation of saplings depends on genetically fixed traits (Kolly & Kupferschmid, 2014). Due to the results obtained by *Pinus* and *Fagus,* it remains unclear to what extent, if at all, the recovery of *Abies* and *Picea* saplings after different intensities of browsing depends on seed origin. The aim of this study was thus to analyze the influence of intraspecific genetic variation in the recovery of two tree species to stress caused by simulated winter browsing and frost damage. The specific research questions were as follows:

- How do *A. alba* and *P. abies* saplings react to simulated browsing and are their reactions dependent on population differences?
- Do different populations recover differently following leader‐shoot loss and thus show genetic variation in sapling recovery traits?
- Are there correlations between climatic and edaphic conditions at the seed source and population differences in the recovery following simulated winter browsing or frost damage?
- Are the genetic differences in the growth of *A. alba* and *P. abies* saplings maintained in the presence of light and heavy browsing?

## 2 MATERIALS AND METHODS

### 2.1 Plant material and experimental setup

In 2009, seeds were sampled from 72 *P. abies*(referred to as *Picea)* and 90 *A. alba*(referred to as *Abies)*seed sources, covering the entire range of climatic conditions suitable for each species in Switzerland (e.g., elevations from 400 to 2,000 m a.s.l., Frank et al., 2017). For each seed source, three parent trees were selected from the same stand but at least 100 m apart to minimize relatedness (Arnold et al., 2010). In April (*A. alba*) and May (*Picea abies*) 2010, seeds from each mother tree (referred to as a “family”) were sown directly into nursery beds at the Swiss Federal Institute for Forest, Snow, and Landscape Research WSL in Birmensdorf, Switzerland.

*Picea* seeds were originally sampled from 92 seed sources, but 20 seed lots consisted of mixed seeds from 10 trees per seed source (Frank et al., 2017) and were omitted from our study. In addition, the seeds from twenty mother trees did not germinate properly, that is, for both species, eight seed sources were represented by seedlings of two families and two seed sources by seedlings of one family only. Throughout the paper, the term “population” refers to individuals whose seeds were collected at the same place of origin. The term “seed source” refers to the location of a population origin.

An extensive common garden (half‐sib progeny) experiment was carried out at the study site Brunnersberg, a former pasture on a south‐facing slope (20%–24% incline) in the Jura Mountains in Switzerland (47°19′35″N, 7°36′42″E, 1,090 m a.s.l.). The site is characterized by a mean annual temperature of 6°C, a mean annual precipitation sum of 1,400 mm (Frank et al., 2017), and a shallow rendzic soil.

In spring 2012, seedlings were transplanted to the study site as bare‐rooted seedlings. The experimental design consisted of 16 plots per species (32 plots total), each plot with six rows of seedlings and a spacing of 30 cm × 40 cm between the seedlings in each plot. Each plot contained one seedling per family, that is, mostly three individuals per population, randomly distributed within the plot. For a detailed description of seed collection, common garden procedures, and the random block design, we refer to Frank et al. (2017). Height, basal diameter, bud phenology, and growth duration were measured in spring and autumn 2013, and values were reported by Frank et al. (2017).

### 2.2 Environmental variables at seed sources

Environmental variables considered at the seed sources included the following: mean annual temperature (*MAT*); mean spring temperature (March – May, *MTsp*); continentality (interannual temperature variance, that is, maximum of warmest month minus minimum of coldest month); average maximum diurnal amplitude of temperature during spring (March – May, *DTAsp*); sum of growing degree days (based on a threshold of 5°C, *DDEG*); average numbers of days during the vegetation season (March – November) with frost (*SFROv*); mean annual precipitation sum (*PREC*); absolute maximum drought (*PREC *< 0.01 mm) period length in summer (June – August, *DRYPsu*); and annual aridity index (*DMI = PREC*/MAT*10 (Martonne, 1926)). All variables were calculated for the period 1931–1960 for each seed source (Frank et al., 2017). Physical and chemical soil properties—including the available water capacity of 1 m soil depth (*AWC*)—were derived from local soil pits that were located within a few meters of one of the three mother trees at each seed source (details see appendix in Frank et al., 2017).

### 2.3 Simulated browsing treatment

On 23 March 2015, before budburst, the five‐year‐old saplings were clipped to simulate a single winter browsing event by roe deer (light clipping) and red deer (heavy clipping). The treatment was applied plot‐wise. For each species, light clipping was applied to six randomly selected plots, while five plots were treated with heavy clipping. Light clipping meant that only the uppermost buds of the leader shoot were removed using pruning shears. For heavy clipping, the annual leader shoot was removed until the remaining shoot segment was 1 cm long. As saplings of *Abies*are more heavily browsed by ungulates than *Picea* saplings*,*heavy clipping of *Abies*also included branch clipping. All vertically growing annual shoots of *Abies* formed in 2014 were cut to 1 cm and all 2014 branches to 2 cm. All older branches of *Abies* were shortened, including 1 cm of the oldest increment; for example for 2012 side shoots, the whole increment from 2014 and 2013 plus 1 cm of the 2012 increment was cut.

### 2.4 Frost damage

In all plots, several saplings had been damaged by late frost events in spring 2013 and 2014. The distribution of the frost events was not even between the plots. However, apart from the exceptions mentioned above, three seedlings of each seed source were planted in every plot, and the position within the plot was randomized. Thus, we assumed no bias of plot position. Frost that affected the leader shoots had chilled the newly formed shoot ends of saplings, causing the young leader shoot to die. This dead shoot remained visible for months as brownish, withered tissue. In cases where a damaged *Abies* was situated within a plot with clipping, it was only clipped at its leader shoot if a clear new leader shoot had already formed by spring 2015 (27% of the damaged saplings). For “damaged” *Abies* growing in plots with heavy clipping, the same branch clipping procedure was applied as for undamaged saplings. The “damaged” *Picea*were not clipped in the 71 cases where they had no new leader shoot in spring 2015, whereas the other 128 “damaged” *Picea*were lightly or heavily clipped on the new leader shoot.

### 2.5 Trait assessment

The growth and quality of saplings were assessed before and one to two vegetation seasons after clipping (Supporting Information Table S1 and Table S2). Sapling height was measured as the vertical distance from the ground surface to the tip of the leader shoot (height) or to the highest point of the tree regardless of whether this was a leader shoot or a branch (tree height). The annual height increment of the leader shoot was measured to an accuracy of 0.5 cm. Measurements of stem diameter were taken 2 cm above the soil surface using electronic calipers (Type M‐150, MBFZ toolcraft GmbH, Georgensgmünd, Germany). In February 2017, all *Abies*were cut 2 cm above the soil surface and their fresh weight was determined immediately with a hanging scale (Kern HDBH 5K5N) with a resolution of 5 g.

In order to estimate dry weight, 50 saplings of each species were harvested from two control plots at the end of the experiment in February 2017. Each of these 100 saplings was cut 2 cm above the soil surface, placed in a paper bag, oven dried for 75 hr (until mass constancy) at 70°C, and weighed at a resolution of 1 mg. Aboveground biomass in 2016 was estimated for *Abies*using an allometric function relating fresh to dry weight (*R* ^2^: 0.9953, *p* < 2.2e‐16). For *Abies*biomass in 2014, a linear regression model for dry weight in 2016 as a function of diameter in 2016 was applied (*R* ^2^: 0.8927, *p* < 2.2e‐16), that is, ln(dry weight 2016) ≈ −2.5386 + 2.4361*ln(diameter 2016), and the values for 2014 were predicted using the diameters in 2014. For *Picea*, the linear regression model for dry weight in 2016 had a much better fit if height in 2016 (*R* ^2^: 0.7607, *p* < 1.962e‐15) was included as an explanatory variable, that is, ln(dry weight 2016) ≈ −2.3223 + 2.2002*ln(diameter 2016) + 0.1791*ln(height 2016). Biomass in 2014 and 2016 was then predicted for all *Picea* saplings using their respective diameter and height measurements.

A total of 18 ordinal traits were assessed. The leader shoots before (2014) and after clipping (2015, 2016), the branches in the uppermost whorl on the highest leader shoot in 2014 and 2016, and the visible buds on the leader shoot in 2014 were all counted. In 2016, stem form and crown form were assessed. We classified stem form as “straight” (deviation from vertical line ≤22.5°), “bent” (deviation 22.5–45°), or “severely bent” (deviation>45°). Crown form was classified using five levels ranging from optimal (1) to low quality (5; a detailed field guide will be made available on EnviDat). In addition, the vitality of living saplings in 2016 was assigned to one of five classes ranging from vital (0) to the presence of several completely withered branches (4).

In autumn 2016, we recorded how the sapling had reacted to leader loss due to frost damage or clipping by evaluating the “location of reaction,” the “reaction type” and the “time lag” of the reaction. The “location of reaction” had three levels: reaction out of “uppermost shoot whorl,” reaction out of “lower shoot whorl,” and “no reaction.” There were six “reaction types”: (a) production of a “new distal shoot” out of a bud on the stem or on the remaining leader shoot pieces, (b) production of a “new basal shoot” out of a bud on a whorl, (c) “flagging” of an existing internodal side shoot, (d) “flagging” of an existing whorl shoot, (e) use of an already vertically growing or bending upward of an “epicormic shoot” that is, a shoot that developed before the stress but was younger than the regular whorl shoots (preventitious shoots after Gruber, 1994), and (f) “no reaction.” The time lag of the reaction after clipping was evaluated as: “0” = clear new leader shoot (longer than 0.5 cm) formed in the first vegetation season after leader loss, “1” = new leader shoot formed in 2016, and “2” = no new leader shoot formed until the end of the second growing season. Likewise, the time lag of the reaction after frost damage (0–4 years) was noted.

We recorded whether the saplings made no second flush at the 2016 leader shoot (0), a second flush with bud dormancy (1), a second flush without bud dormancy (2), or a second flush with a combination of dormancy and no dormancy (3). Finally, we assessed whether the saplings had other leader damages, like insect browsing or damage caused by site maintenance, and excluded these few saplings from further analysis (*N* = 48 for *Abies*and *N* = 38 for *Picea*).

### 2.6 Data analysis

Analysis of variance was performed using different functions for the three different data types. For the continuous traits, we applied a linear mixed‐effects model using the *lmer* function (package “lme4”; Bates, Maechler, Bolker, & Walker, 2015) in R version 3.3.3 (R Core Team, 2017). For the ordinal traits, we applied a cumulative link mixed model using the R function *clmm* (package “ordinal”; Christensen, 2015). For the binary trait “frost damage occurrence,” we applied a generalized linear mixed‐effects model using the R function *glmer* (package “lme4,” binomial model, link = “logit,” optimizer = “bobyqa”). An intercept, the treatment (4 levels: no, light and heavy clipping, frost damage) and a covariate (see below) were included as fixed effects and plot, population, family, and the interaction between population and treatment were included as random effects in the mixed‐effects models. The interaction between population and treatment was not significant and including it resulted in model convergence failure. This interaction was thus only retained in the model for the continuous traits of *Abies*. The covariate (omitted for the binary trait “frost damage”) was either height or diameter in 2012, and it was included to account for differences already apparent in the nursery and differences caused by different planting depths.

To test for the significance of all factors in our mixed‐effects model, likelihood ratio tests were used that compared the full model to the same model without the terms that should be tested (R function *ANOVA*). If the treatment was significant at *p* < 0.01, a Tukey post hoc test was used to distinguish between the effects of the four treatment levels, that is, the three clipping levels and (frost) “damaged” (R function *glht*, package “multcomp”; (Hothorn, Bretz, & Westfall, 2008)).

In cases where there was a significant population effect (*p* < 0.01) in the mixed‐effects model, we calculated Pearson correlations using the R function *rcorr* (package “Hmisc”) between population random effects obtained from the analyses of variance (R function *ranef*, package “lme4”) and site‐specific environmental variables at the seed source. If a correlation was significant at *p* < 0.01, we used robust line fitting (R function *line*) to analyze the linear relationships between the population random effects of the respective trait with this site‐specific environmental variable.

To investigate the effect of “reaction type” on the height of the saplings in 2016, similar linear mixed‐effects models were fitted but “reaction type” was added as a fixed effect instead of treatment. These models were fitted separately for lightly and heavily clipped saplings (using the R function *lmer*) and a Tukey post hoc test was used to distinguish between the effects of the “reaction types”.

## 3 RESULTS

### 3.1 *Abies alba*

**Figure 1:**
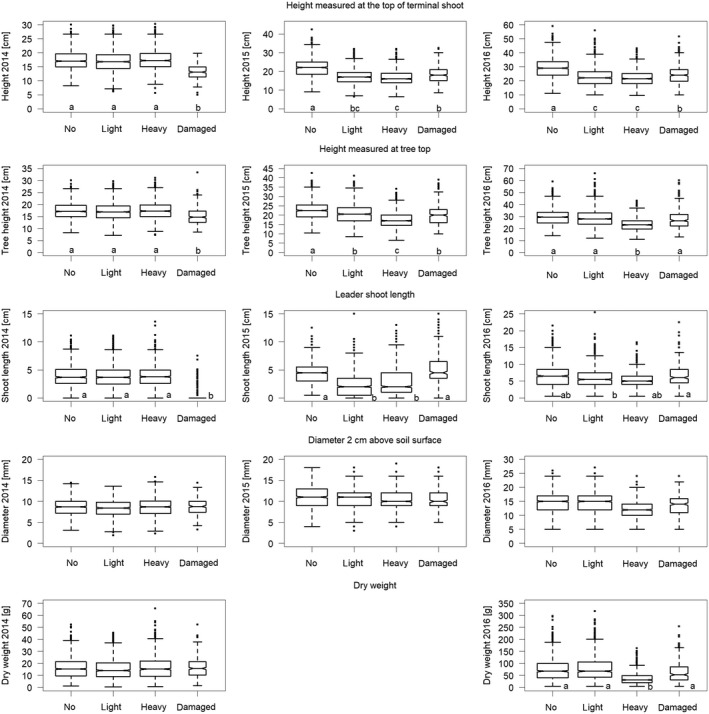
Growth traits of *Abies alba* saplings before (2014), one (2015) and two (2016) vegetation periods after simulated browsing; “no” = not clipped and not damaged, “light” = only apical bud removal on the terminal shoot but no further damage, “heavy” = leader shoot and all twigs clipped but no further damage, “damaged” = frost damage before clipping. Lower case letters indicate significant differences at *p* ≤ 0.05 between the treatments in the Tukey post hoc tests (for cases where the variable “treatment” was significant at *p* < 0.01 in the linear mixed‐effects models; see Table 1). Median (bold line), first, and third quartile (bottom and top of box), quartile ±1.5 * interquartile range (whiskers) and individual points more extreme in value (circles) were drawn using the “boxplot” function in default R code. The width of the boxes represents the number of trees within the various categories

Frost damage and clipping both resulted in reduced height growth of *Abies*in the first year after the stress but not in the following years (Figure 1). No significant difference was found between light and heavy clipping. Height was reduced for at least two (clipping) and up to four (frost damage) vegetation seasons in comparison with unclipped and undamaged trees. Height at the tree top was greater for lightly than for heavily clipped trees, as branches of lightly clipped *Abies*were often higher than the new leader shoot. Basal diameter was affected neither by frost damage nor by clipping (Table 1). The diameter increment from 2014 to 2016 was significantly smaller only for heavily clipped *Abies*. Aboveground fresh weight was also only reduced after heavy clipping (Figure 1).

**Table 1:** Results of the linear mixed‐effects models for *Abies alba* saplings and their growth traits

Trait	Model details					Random effects					Fixed effects					*p* values of full models						p values of post hoc tests					
	TF	Covariate	*N* tot	Mean	*SD*	Block	Pop	Family	T x Pop	Residual	Intercept	Covariate	L	H	D	Block	Pop	Family	T x Pop	Covariate	T	L‐no	H‐no	D‐no	H‐L	D‐L	D‐H
Diameter 2014	no	D12	3,745	8.5	2.1	0.291	0.107	0.105	0.033	2.128	2.714	1.865	0.056	0.125	0.219	***<0.001***	***<0.001***	***<0.001***	0.070	***<0.001***	0.145	1.000	1.000	0.822	1.000	1.000	1.000
Diameter 2015	no	D12	3,533	10.6	2.5	0.393	0.148	0.180	0.092	3.328	3.862	2.204	−0.106	−0.236	−0.183	***<0.001***	***<0.001***	***<0.001***	***0.004***	***<0.001***	*0.046*	1.000	1.000	1.000	1.000	1.000	1.000
Diameter 2016	no	D12	3,426	13.9	3.6	1.412	0.245	0.364	0.131	6.912	5.690	2.712	−0.109	−0.820	−0.220	***<0.001***	***<0.001***	***<0.001***	*0.050*	***<0.001***	0.056	1.000	0.156	1.000	0.253	1.000	0.158
DI	no	D12	3,426	5.1	2.3	0.923	0.028	0.132	0.039	3.224	2.456	0.972	−0.240	−1.009	−0.368	***<0.001***	0.151	***<0.001***	0.200	***<0.001***	***0.003***	1.000	***<0.001***	0.311	***0.009***	1.000	***0.003***
Height 2014	ln	H12	3,745	164.0	1.3	0.002	0.002	0.001	<0.001	0.022	2.168	0.637	−0.002	0.022	−0.236	***<0.001***	***<0.001***	***<0.001***	0.533	***<0.001***	***<0.001***	1.000	1.000	***<0.001***	0.978	***<0.001***	***<0.001***
Height 2015	ln	H12	3,526	179.5	1.3	0.003	0.003	0.001	<0.001	0.031	2.761	0.554	−0.193	−0.233	−0.150	***<0.001***	***<0.001***	*0.024*	0.313	***<0.001***	***<0.001***	***<0.001***	***<0.001***	***<0.001***	0.379	*0.045*	***<0.001***
Height 2016	ln	H12	3,427	235.1	1.3	0.005	0.002	0.002	0.001	0.049	3.659	0.418	−0.209	−0.205	−0.125	***<0.001***	***<0.001***	***<0.001***	*0.023*	***<0.001***	***<0.001***	***<0.001***	***<0.001***	***<0.001***	1.000	***<0.001***	***0.002***
Tree height 2014	ln	H12	3,745	165.7	1.2	0.002	0.002	0.001	<0.001	0.022	2.168	0.640	−0.013	−0.008	−0.116	***<0.001***	***<0.001***	***<0.001***	0.317	***<0.001***	***<0.001***	1.000	1.000	***<0.001***	1.000	***<0.001***	***<0.001***
Tree height 2015	ln	H12	3,526	196.4	1.3	0.006	0.002	0.001	<0.001	0.036	2.635	0.587	−0.081	−0.153	−0.060	***<0.001***	***<0.001***	***0.005***	0.403	***<0.001***	***<0.001***	***0.006***	***<0.001***	*0.012*	*0.018*	1.000	***<0.001***
Tree height 2016	ln	H12	3,427	262.4	1.3	0.008	0.003	0.002	<0.001	0.044	3.617	0.432	−0.042	−0.117	−0.037	***<0.001***	***<0.001***	***<0.001***	0.674	***<0.001***	***0.004***	0.770	***<0.001***	0.494	*0.037*	1.000	***0.001***
Shoot length 2014	sqrt	H12	3,409	3.7	0.2	0.015	0.011	0.008	0.002	0.202	1.540	0.038	0.001	−0.019	−0.277	***<0.001***	***<0.001***	***<0.001***	0.279	***<0.001***	***0.006***	1.000	1.000	*0.011*	1.000	***0.004***	*0.016*
Shoot length 2015	sqrt	H12	3,255	3.2	0.4	0.024	0.013	0.007	0.005	0.311	2.001	0.005	−0.481	−0.502	0.111	***<0.001***	***<0.001***	*0.020*	0.129	0.657	***<0.001***	***<0.001***	***<0.001***	0.221	1.000	***<0.001***	***<0.001***
Shoot length 2016	sqrt	H12	3,367	5.7	0.3	0.034	<0.001	0.012	0.008	0.289	1.822	0.060	−0.140	−0.060	0.037	***<0.001***	*0.010*	***<0.001***	***0.002***	***<0.001***	***<0.001***	0.252	1.000	1.000	1.000	***0.003***	0.431
Biomass 2014	sqrt	D12	3,745	14.9	1.3	0.084	0.030	0.030	0.010	0.629	−2.287	3.502	0.024	0.071	0.111	***<0.001***	***<0.001***	***<0.001***	0.071	***<0.001***	0.174	1.000	1.000	0.994	1.000	1.000	1.000
Biomass 2016	sqrt	D12	3,427	57.0	7.5	0.891	0.194	0.224	0.083	3.828	−3.345	6.371	−0.097	−1.020	−0.363	***<0.001***	***<0.001***	***<0.001***	*0.025*	***<0.001***	***0.001***	1.000	***0.001***	0.475	***0.003***	0.957	***0.008***
Fresh weight 2016	sqrt	D12	3,427	120.3	17.4	2.067	0.451	0.520	0.188	8.870	−5.624	9.699	−0.143	−1.551	−0.544	***<0.001***	***<0.001***	***<0.001***	*0.029*	***<0.001***	***0.001***	1.000	***0.001***	0.504	***0.003***	0.981	***0.007***

Note

*Note*. Model details include the transformation (TF) applied to response variables and covariates, the covariate included (D12 = basal diameter in 2012, H12 = height in 2012), the number of analyzed saplings (N tot), and the mean and standard deviation (SD) of the trait (not transformed). Trait “DI” is the diameter increment measured as diameter in 2016 minus diameter in 2014. For the random effects, the variances are given, and for the fixed effects, the estimated coefficients are given. Population has been cut to Pop and the treatments (T) to "no" for no clipping and not damaged, "L" for light clipping, "H" for heavy clipping and "D" for damaged. p values of the likelihood ratio tests for each variable in the full models (DF = 10) and of the Tukey post hoc tests of the treatment are printed in bold and italics for p ≤ 0.01 and in italics for p ≤ 0.05.

**Figure 2:**
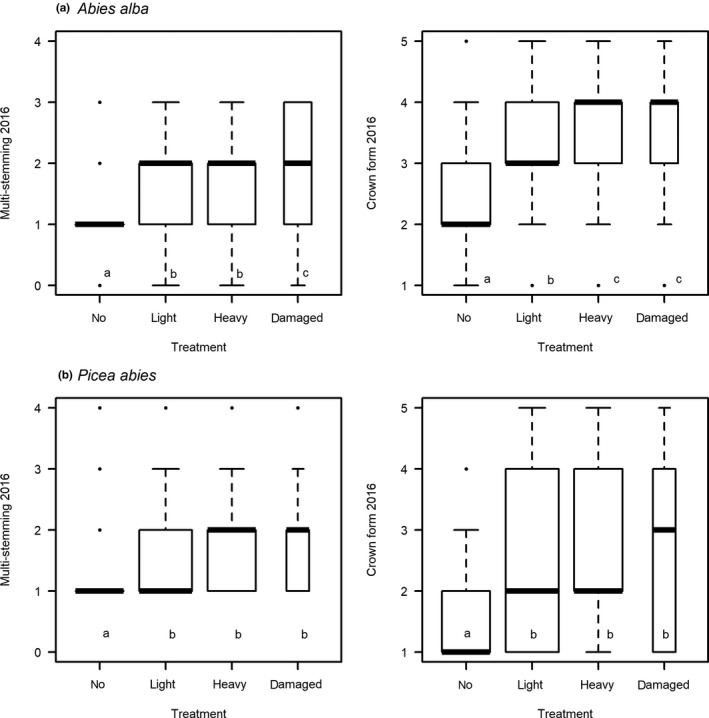
Morphological traits of *Abies* (a) and *Picea*(b) saplings two vegetation periods after simulated browsing; “no” = not clipped, “light” = only bud removal, and “heavy” = leader shoot clipped and in the case of *Abies alba* also all twigs clipped, “damaged” = frost damage before clipping. Lower case letters indicate significant differences at *p* ≤ 0.05 between the treatments in the Tukey post hoc tests (for cases where *p* ≤ 0.001 in the mixed‐effect models; see Tables 2 and 5)

Quality decreased with the intensity of clipping (Figure 2a), in particular crown form, and thus, overall quality was negatively affected by clipping and frost damage. Multistemmed saplings were frequent after clipping and even more so after frost damage. Stem form and vitality were, in contrast, not affected by either stress (Table 2). The number of branches in the uppermost whorl was reduced by frost damage in 2014 (whorl shoots 13/14) but not any more in 2016 (whorl shoots 15/16). In 2016, the number of whorl shoots 15/16 was reduced after clipping.

**Table 2:** Results of the cumulative link mixed model for *Abies alba* saplings and their quality traits

Trait	*N*	Random effects			Fixed effects				*p* values of full models					p values of post hoc tests					
		Block	Pop	Family	Covariate	L	H	D	Block	Population	Family	Covariate	T	L‐no	H‐no	D‐no	H‐L	D‐L	D‐H
Multistemming 2014	3,672	<0.001	<0.001	<0.001	−0.001	−0.117	−0.409	−6.753	1.000	1.000	1.000	0.851	***<0.001***	1.000	1.000	***<0.001***	1.000	***<0.001***	***<0.001***
Multistemming 2015	3,527	0.087	0.028	0.012	−0.004	1.484	2.465	2.554	***<0.001***	0.158	0.674	***0.008***	***<0.001***	***<0.001***	***<0.001***	***<0.001***	***<0.001***	***<0.001***	1.000
Multistemming 2016	3,427	0.103	0.014	0.002	−0.002	2.274	2.504	3.038	***<0.001***	0.525	0.964	0.163	***<0.001***	***<0.001***	***<0.001***	***<0.001***	1.000	***<0.001***	***0.007***
Quality 2016	3,422	0.190	0.001	0.032	−0.013	2.847	4.687	4.296	***<0.001***	0.950	0.340	***<0.001***	***<0.001***	***<0.001***	***<0.001***	***<0.001***	***<0.001***	***<0.001***	0.184
Crown form 2016	3,422	0.255	<0.001	0.060	−0.013	3.089	5.133	4.669	***<0.001***	0.991	0.068	***<0.001***	***<0.001***	***<0.001***	***<0.001***	***<0.001***	***<0.001***	***<0.001***	0.086
Stem form 2016	3,422	0.344	0.051	0.122	0.003	0.480	0.324	0.399	***<0.001***	0.204	*0.010*	0.110	0.244	0.348	1.000	0.359	1.000	1.000	1.000
Vitality 2016	3,422	0.212	0.029	0.115	0.004	0.033	0.101	−0.173	***<0.001***	0.345	***0.004***	*0.029*	0.372	1.000	1.000	1.000	1.000	1.000	0.840
Whorl shoots 2013/2014	3,649	0.060	0.035	0.224	0.016	−0.071	0.081	−1.035	***<0.001***	0.282	***<0.001***	***<0.001***	***<0.001***	1.000	1.000	***<0.001***	1.000	***<0.001***	***<0.001***
Whorl shoots 2015/2016	3,368	0.219	0.097	0.091	0.001	−1.773	−1.055	0.021	***<0.001***	***0.002***	***0.007***	0.692	***<0.001***	***<0.001***	***<0.001***	1.000	*0.010*	***<0.001***	***<0.001***
Buds on leader shoot 2014	3,375	0.223	0.128	0.131	−0.001	0.025	0.220	−0.218	***<0.001***	***<0.001***	***0.000***	0.541	0.586	1.000	1.000	1.000	1.000	1.000	1.000
Reaction type (clipping)	2,187	0.008	0.015	0.047	−0.002	NA	0.668	NA	0.395	0.649	0.359	0.229	***<0.001***	NA	NA	NA	***<0.001***	NA	NA
Reaction location (clipping)	2,187	<0.001	<0.001	0.085	−0.005	NA	−0.777	NA	1.000	1.000	0.359	0.081	***<0.001***	NA	NA	NA	***<0.001***	NA	NA
Time lag (clipping)	2,187	0.222	0.183	0.102	0.009	NA	−0.609	NA	***<0.001***	***0.002***	0.122	***<0.001***	0.063	NA	NA	NA	*0.044*	NA	NA
Reaction type (damage)	324	NA	<0.001	3.859	0.002	−0.299	−0.421	NA	NA	0.985	***0.007***	0.793	0.653	1.000	1.000	NA	1.000	NA	NA
Reaction location (damage)	324	NA	<0.001	0.020	−0.006	0.790	1.051	NA	NA	1.000	0.945	0.322	***0.005***	0.065	***0.007***	NA	1.000	NA	NA
Time lag (damage)	318	NA	<0.001	<0.001	0.016	−0.584	−0.630	NA	NA	1.000	1.000	***0.008***	0.076	0.163	0.130	NA	1.000	NA	NA

Note

*Note*. The number of analyzed saplings is given (N). The covariate was height in 2012. Other details as in Table 1.

**Figure 3:**
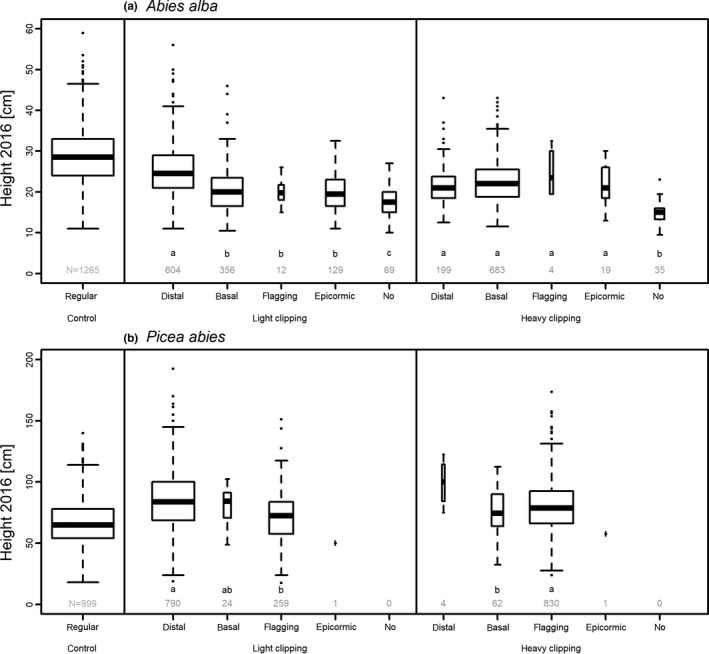
Relationship between height in 2016 and reaction type for *Abies* (a) and *Picea* (b) saplings that were not clipped (control), lightly clipped or heavily clipped, excluding all frost damaged trees. The number of observations (*N*) per reaction type are shown in gray. Lower case letters indicate significant differences at *p* ≤ 0.05 between the reaction types within each treatment in the Tukey post hoc tests

“Reaction type” and “location of reaction” differed between the treatments (Table 2), and this resulted in significant differences in tree height in the year 2016 (Figure 3a). After light clipping, *Abies* mostly reacted with shoots formed out of distal buds on the remaining stem piece of the 2014 height increment and were the tallest in the second vegetation seasons after clipping. Heavily clipped *Abies*most often used basal buds of the uppermost shoot to form a new leader shoot, followed by distal buds on the height increment of 2013 (i.e. had another “location of reaction”; Figure 3a). *Abies*with no reaction through the end of the experiment were the smallest after both light and heavy clipping. The time lag between clipping and the formation of a clear new leader shoot was independent of browsing intensity (Table 1). Of all *Abies* saplings, 28.4% showed a reaction time lag of one year, that is, they mostly formed a new visible bud without elongation growth. About 4.9% of the saplings (108 of the 2,187 saplings) still had no leader shoot at the end of the experiment.

**Figure 4:**
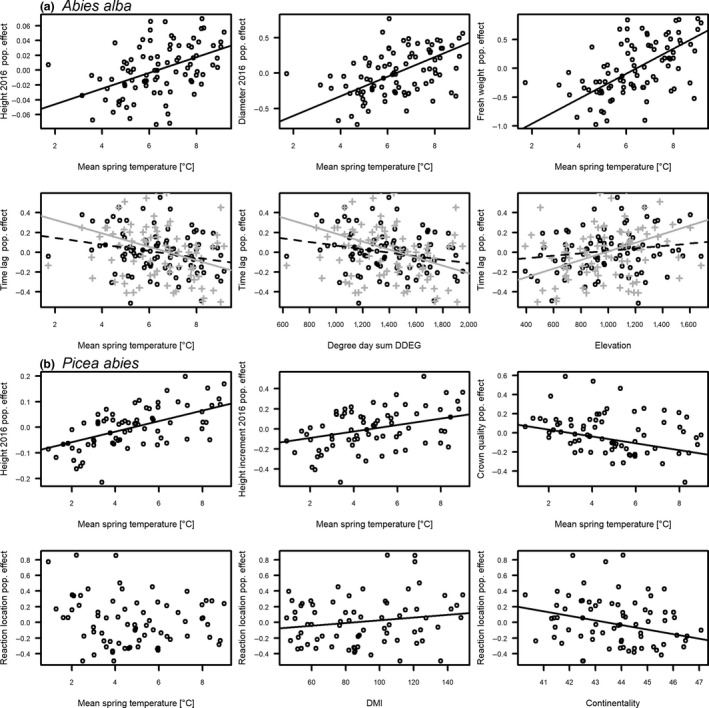
Linear relationships of population effects to environmental conditions at seed source for 90 *Abies* (a) and 72 *Picea* (b) populations from Switzerland. Note that the population effects are not equally scaled. Regression lines are displayed only for significant (*p* < 0.001) relationships in the linear mixed‐effect models (Tables 1, 2 and 4, 5) and in the Pearson correlations (Tables 3 and 6). Lines are drawn using robust line fitting (R function *line*). In the case of *Abies alba* and time lag, regression lines are shown separately for lightly (dashed black) and heavily (solid gray) clipped saplings

Population differences were significant for all growth traits except the diameter increment (Table 1), but hardly ever for quality variables (Table 2). Diameter, height, height increment, and aboveground biomass all decreased with decreasing temperature (*MAT* and *MTsp*; Figure 4a), *DDEG*and continentality at the seed source (Table 3). Therefore, these traits also decreased with increasing elevation, geographical longitude, and hill slope. Fewer frosts (*SFROv*) and a drier climate at the seed source (*PREC, DryPsu, DMI*) corresponded to higher values of most growth traits. Soil properties had less influence than the climate at the seed source, but less sand, more clay and a smaller C/N ratio were correlated with higher values of many growth traits (Table 3). The number of buds on the leader shoots formed in 2014 increased with increasing temperature at the seed source (Table 3) and was positively correlated with diameter and height in 2014 (Pearson correlation coefficient (corr) of 0.6).

**Table 3:** Trait–environment relationships between sapling trait provenance effects and environmental variables for 90 provenances of *Abies alba*, displayed using Pearson correlation coefficients

Trait	Geography and topography				Soil properties							Temperature						Precipitation		
	Elevation	Latitude	Longitude	Slope	Sand	Clay	N_tot	C_tot	C_N	pH of top layer	AWC	MAT	MTSp	DTAsp	cont	DDEG5	SFROv	PREC	DRYPsu	DMI
Diameter 2014	***−0.464***	***0.475***	***−0.290***	***−0.331***	***−0.158***	***0.205***	0.101	0.066	***−0.188***	0.110	−0.091	***0.403***	***0.408***	***0.465***	***0.322***	***0.427***	***−0.200***	***−0.198***	***0.482***	***−0.282***
Diameter 2015	***−0.424***	***0.420***	***−0.323***	***−0.354***	−0.134	***0.218***	0.146	0.115	***−0.182***	0.109	−0.089	***0.390***	***0.385***	***0.447***	***0.298***	***0.398***	***−0.204***	***−0.098***	***0.442***	***−0.199***
Diameter 2016	***−0.453***	***0.376***	***−0.321***	***−0.340***	−0.110	***0.157***	0.134	0.112	−0.154	0.081	−0.055	***0.448***	***0.440***	***0.400***	***0.222***	***0.451***	***−0.267***	***−0.136***	***0.437***	***−0.243***
Height 2014	***−0.469***	***0.556***	***−0.176***	***−0.283***	***−0.199***	***0.239***	−0.002	−0.033	***−0.158***	0.124	−0.115	***0.357***	***0.375***	***0.437***	***0.382***	***0.401***	−0.132	***−0.298***	***0.491***	***−0.346***
Height 2015	***−0.518***	***0.557***	***−0.277***	***−0.335***	***−0.225***	***0.269***	0.062	0.038	−0.148	0.140	−0.080	***0.431***	***0.439***	***0.469***	***0.373***	***0.465***	***−0.174***	***−0.313***	***0.557***	***−0.378***
Height 2016	***−0.516***	***0.581***	***−0.302***	***−0.381***	***−0.275***	***0.311***	0.047	0.020	***−0.214***	0.134	−0.034	***0.425***	***0.431***	***0.461***	***0.357***	***0.464***	***−0.217***	***−0.256***	***0.564***	***−0.323***
Tree height 2014	***−0.479***	***0.576***	***−0.173***	***−0.302***	***−0.206***	***0.251***	−0.006	−0.045	***−0.177***	0.118	−0.126	***0.370***	***0.388***	***0.453***	***0.402***	***0.419***	−0.152	***−0.313***	***0.507***	***−0.359***
Tree height 2015	***−0.506***	***0.591***	***−0.225***	***−0.392***	***−0.233***	***0.237***	0.023	0.001	−0.117	0.074	−0.069	***0.384***	***0.398***	***0.453***	***0.395***	***0.437***	***−0.171***	***−0.247***	***0.561***	***−0.305***
Tree height 2016	***−0.508***	***0.596***	***−0.236***	***−0.402***	***−0.252***	***0.279***	0.016	−0.010	***−0.193***	0.076	−0.001	***0.414***	***0.428***	***0.451***	***0.360***	***0.468***	***−0.236***	***−0.254***	***0.543***	***−0.316***
Shoot length 2014	***−0.436***	***0.461***	***−0.180***	***−0.235***	−0.094	***0.163***	−0.013	−0.033	−0.053	0.132	−0.126	***0.367***	***0.377***	***0.347***	***0.291***	***0.400***	***−0.176***	***−0.338***	***0.459***	***−0.374***
Shoot length 2015	***−0.361***	***0.264***	***−0.385***	***−0.230***	−0.085	0.151	0.091	0.089	−0.004	***0.223***	−0.036	***0.351***	***0.331***	***0.275***	0.149	***0.338***	***−0.256***	***−0.222***	***0.452***	***−0.274***
Shoot length 2016	***−0.202***	***0.242***	−0.148	***−0.213***	−0.132	***0.192***	0.034	0.044	−0.104	0.103	−0.013	0.154	***0.159***	***0.186***	***0.198***	***0.175***	***−0.160***	−0.062	***0.219***	−0.090
Fresh weight 2016	***−0.582***	***0.501***	***−0.241***	***−0.374***	−0.137	0.150	0.070	0.070	−0.132	0.016	−0.001	***0.532***	***0.542***	***0.478***	***0.263***	***0.560***	***−0.288***	***−0.190***	***0.447***	***−0.311***
Biomass 2014	***−0.467***	***0.480***	***−0.287***	***−0.341***	−0.154	***0.206***	0.106	0.069	***−0.194***	0.115	−0.090	***0.410***	***0.413***	***0.467***	***0.321***	***0.433***	***−0.209***	***−0.196***	***0.488***	***−0.282***
Biomass 2016	***−0.583***	***0.502***	***−0.241***	***−0.373***	−0.136	0.150	0.070	0.070	−0.133	0.017	−0.002	***0.533***	***0.543***	***0.480***	***0.265***	***0.561***	***−0.288***	***−0.193***	***0.448***	***−0.314***
Whorl shoots 2015/2016	***−0.490***	***0.326***	***−0.339***	***−0.268***	−0.039	0.108	−0.020	−0.021	−0.049	0.055	0.028	***0.414***	***0.409***	0.384	***0.160***	***0.436***	−0.288	−0.131	***0.407***	***−0.214***
Buds on leader shoot 2014	***−0.422***	***0.315***	***−0.310***	***−0.248***	−0.084	***0.173***	0.045	0.055	0.026	***0.168***	−0.083	***0.430***	***0.423***	0.344	***0.216***	***0.433***	−0.237	***−0.364***	***0.377***	***−0.416***
Time lag (clipping)	***0.340***	***−0.219***	***0.302***	***0.252***	0.084	−0.097	−0.106	−0.097	0.062	−0.094	0.039	***−0.308***	***−0.294***	−0.177	−0.010	***−0.306***	0.266	0.068	***−0.311***	*0.131*

Note

*Note*. Significant correlations (*p* < 0.01) are highlighted in bold italics.

Neither the “reaction type” nor the “location of reaction” differed among the provenances, but the time until a new leader was formed varied significantly among the *Abies* populations (Table 2). The reaction time lag increased with decreasing temperature at the seed source, decreasing sum of growing degree days, and increasing elevation (Figure 4a). The time lag was negatively correlated with all growth traits (corr between −0.33 and −0.71, depending on trait and year).

Frost damage affected 9.5% (324) of the 3,427 *Abies*still alive in autumn 2016. Of these frost damaged *Abies*, 67.3% had a time lag in their reaction of forming a clear new shoot of one year, ten saplings had a time lag of two years and ten of ≥three years. The large majority (> 90%) of *Abies*reacted to frost damage by forming a new leader shoot out of a basal bud on the uppermost whorl (Supporting Information Figure S1), independent of seed source (Table 2). However, seed source nevertheless seemed to play a role in the reaction to frost, as the “reaction type” after frost varied among the families (Table 2). Additionally, in the mixed‐effects models for the binary trait “frost damage occurrence,” family tended to show differences (*p* = 0.074). Further, “frost damage occurrence” correlated positively with the C/N ratio (corr = 0.142), temperature (corr with MAT = 0.156 and with MTSp = 0.146), and DDEG (corr = 0.134), but negatively with precipitation (corr = −0.18) and DMI at the seed source (corr = −0.19).

### 3.2 *Picea abies*

**Figure 5:**
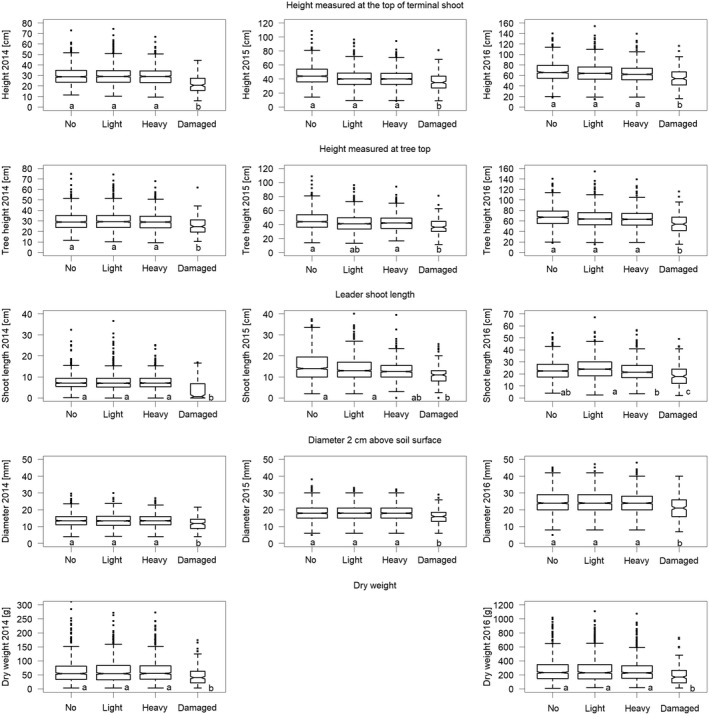
Growth traits of *Picea abies*saplings before (2014), one (2015) and two (2016) vegetation periods after simulated browsing; “no” = not clipped, “light” = only apical bud removal on the terminal shoot, “heavy” = leader shoot clipped, “damaged” = frost damage before clipping. Letters refer to significant differences at *p* ≤ 0.05 between the treatments in the Tukey post hoc tests. For plotting details, see Figure 1

*Picea*grew more than twice as fast as *Abies*saplings in both height and stem diameter (Figure 1 vs. Figure 5). Frost damage but not clipping resulted in a significant reduction of height growth, height, biomass, and basal diameter of *Picea*for at least three to four vegetation seasons (Figure 5). Nevertheless, lightly clipped *Picea*had larger height increments in the year 2016 compared to heavily clipped saplings (Table 4).

**Table 4:** Results of the linear mixed‐effects models for *Picea abies* saplings and their growth traits

**Trait**	Model details					Random effects				Fixed effects					*p* values of full models					p values of post hoc tests					
	TF	Covariate	*N* tot	Mean	*SD*	Block	Pop	Family	Residual	Intercept	Covariate	L	H	D	Block	Pop	Family	Covariate	T	L‐no	H‐no	D‐no	H‐L	D‐L	D‐H
Diameter 2014	no	D12	3,079	13.5	3.7	0.311	0.572	0.110	4.592	3.799	2.610	−0.176	0.327	−0.784	***<0.001***	***<0.001***	*0.010*	***<0.001***	***<0.001***	1.000	1.000	***0.004***	0.373	*0.031*	***<0.001***
Diameter 2015	no	D12	3,065	18.1	4.5	0.461	0.952	0.307	7.685	7.238	2.956	−0.347	0.071	−1.510	***<0.001***	***<0.001***	***<0.001***	***<0.001***	***<0.001***	1.000	1.000	***<0.001***	1.000	***<0.001***	***<0.001***
Diameter 2016	no	D12	3,063	24.3	6.5	0.601	2.877	0.591	17.209	9.696	3.950	−0.380	0.227	−1.738	***<0.001***	***<0.001***	***<0.001***	***<0.001***	***<0.001***	1.000	1.000	***<0.001***	0.994	***0.003***	***0.000***
DI	no	D12	3,063	10.8	3.7	0.327	0.847	0.281	9.355	5.640	1.409	−0.234	−0.065	−0.972	***<0.001***	***<0.001***	***0.002***	***<0.001***	***0.002***	1.000	1.000	***0.008***	1.000	0.065	*0.016*
Height 2014	ln	H12	3,079	275.9	1.4	0.003	0.005	0.001	0.028	2.842	0.559	0.003	−0.021	−0.227	***<0.001***	***<0.001***	***<0.001***	***<0.001***	***<0.001***	1.000	1.000	***<0.001***	1.000	***<0.001***	***<0.001***
Height 2015	ln	H12	3,066	395.4	1.4	0.004	0.007	0.001	0.043	3.366	0.532	−0.061	−0.049	−0.121	***<0.001***	***<0.001***	***<0.001***	***<0.001***	***<0.001***	0.173	0.531	***<0.001***	1.000	*0.033*	*0.011*
Height 2016	ln	H12	3,063	620.2	1.3	0.003	0.008	0.002	0.035	4.216	0.450	−0.044	−0.041	−0.120	***<0.001***	***<0.001***	***<0.001***	***<0.001***	***<0.001***	0.461	0.676	***<0.001***	1.000	***0.001***	***0.001***
Tree height 2014	ln	H12	3,079	281.5	1.4	0.003	0.006	0.001	0.026	2.819	0.563	0.009	−0.001	−0.055	***<0.001***	***<0.001***	***<0.001***	***<0.001***	***<0.001***	1.000	1.000	*0.016*	1.000	***0.001***	*0.019*
Tree height 2015	ln	H12	3,066	411.6	1.3	0.003	0.006	0.001	0.028	3.498	0.511	−0.056	−0.031	−0.098	***<0.001***	***<0.001***	***<0.001***	***<0.001***	***<0.001***	0.113	1.000	***<0.001***	1.000	0.115	***0.003***
Tree height 2016	ln	H12	3,063	620.2	1.3	0.003	0.008	0.002	0.035	4.217	0.449	−0.043	−0.041	−0.118	***<0.001***	***<0.001***	***<0.001***	***<0.001***	***<0.001***	0.496	0.683	***<0.001***	1.000	***0.001***	***0.001***
Shoot length 2014	sqrt	H12	2,992	7.1	0.4	0.027	0.018	0.012	0.327	2.106	0.049	−0.035	−0.071	−0.317	***<0.001***	***<0.001***	***<0.001***	***<0.001***	***<0.001***	1.000	1.000	***<0.001***	1.000	***0.001***	***0.006***
Shoot length 2015	sqrt	H12	3,064	13.3	0.5	0.035	0.046	0.016	0.340	2.586	0.094	−0.130	−0.146	−0.298	***<0.001***	***<0.001***	***<0.001***	***<0.001***	***<0.001***	0.641	0.484	***<0.001***	1.000	*0.041*	0.123
Shoot length 2016	sqrt	H12	3,063	22.3	0.8	0.012	0.057	0.031	0.563	3.217	0.123	0.087	−0.101	−0.360	***<0.001***	***<0.001***	***<0.001***	***<0.001***	***<0.001***	1.000	0.924	***<0.001***	*0.034*	***<0.001***	***0.001***
Second flush length 2016	sqrt	H12	632	5.9	0.5	0.027	0.002	0.043	0.378	2.325	<0.001	0.145	0.029	−0.241	***<0.001***	0.843	***0.002***	0.993	0.066	1.000	1.000	0.857	1.000	0.051	0.545
Biomass 2014	sqrt	D12	3,079	56.1	6.0	0.136	0.245	0.048	1.908	−5.198	6.636	−0.098	0.199	−0.509	***<0.001***	***<0.001***	***0.007***	***<0.001***	***<0.001***	1.000	1.000	***0.004***	0.547	*0.021*	***<0.001***
Biomass 2016	sqrt	D12	3,063	235.3	23.1	0.370	1.522	0.337	9.059	−6.531	11.496	−0.331	0.085	−1.337	***<0.001***	***<0.001***	***<0.001***	***<0.001***	***<0.001***	1.000	1.000	***<0.001***	1.000	***0.003***	***<0.001***

Note

*Note*. Details as in Table 1.

Most quality traits were significantly reduced for the clipped and “frost damaged” *Picea*. Multistemming very rarely occurred for control saplings, was frequent for lightly clipped saplings, and was common for heavily clipped and frost damaged saplings (Figure 2b). Crown form had a reduced quality in clipped and frost damaged saplings, irrespective of the cause of leader loss. Stem form and sapling vitality were not affected by clipping or frost damage (Table 5).

**Table 5:** Results of the cumulative link mixed model for *Picea abies* saplings and their quality traits

Trait	*N*	Random effects			Fixed effects				*p* values of full models					p values of post hoc tests					
		Block	Pop	Family	Covariate	L	H	D	Block	Pop	Family	Covariate	T	L‐no	H‐no	D‐no	H‐L	D‐L	D‐H
Multistemming 2014	3,074	<0.001	0.220	<0.001	−0.005	0.351	−0.381	−7.916	1.000	0.261	1.000	0.050	***<0.001***	1.000	1.000	***<0.001***	1.000	***<0.001***	***<0.001***
Multistemming 2015	3,066	0.065	0.011	0.113	−0.004	1.188	1.283	1.067	***<0.001***	0.693	***0.003***	***<0.001***	***<0.001***	***<0.001***	***<0.001***	***<0.001***	1.000	1.000	1.000
Multistemming 2016	3,063	0.062	0.069	0.194	−0.007	1.008	1.278	1.268	***<0.001***	0.109	***<0.001***	***<0.001***	***<0.001***	***<0.001***	***<0.001***	***<0.001***	0.669	0.903	1.000
Quality 2016	3,063	0.032	0.050	0.137	−0.007	1.072	1.266	1.342	***0.006***	0.145	***0.001***	***<0.001***	***<0.001***	***<0.001***	***<0.001***	***<0.001***	0.941	0.598	1.000
Crown form 2016	3,063	0.044	0.092	0.074	−0.007	1.452	1.759	1.692	***<0.001***	***0.002***	*0.026*	***<0.001***	***<0.001***	***<0.001***	***<0.001***	***<0.001***	0.198	0.789	1.000
Stem form 2016	3,063	0.092	<0.001	<0.001	0.001	1.136	2.404	3.026	0.073	1.000	1.000	0.656	***<0.001***	0.130	***<0.001***	***<0.001***	***<0.001***	***<0.001***	0.161
Vitality 2016	3,063	0.175	<0.001	0.196	0.003	0.333	0.208	0.816	***<0.001***	1.000	***0.009***	***0.007***	*0.012*	1.000	1.000	***0.009***	1.000	0.268	0.087
Whorl shoots 2013/2014	3,053	0.015	0.265	0.091	0.003	0.013	0.009	−1.416	0.052	***<0.001***	***0.003***	***<0.001***	***<0.001***	1.000	1.000	***<0.001***	1.000	***<0.001***	***<0.001***
Whorl shoots 2015/2016	3,063	0.090	0.156	0.168	0.004	−1.267	−1.087	−1.098	***<0.001***	***<0.001***	***<0.001***	***<0.001***	***<0.001***	***<0.001***	***<0.001***	***<0.001***	1.000	1.000	1.000
Buds on leader shoot 2014	2,965	1.144	0.048	0.203	−0.001	−0.192	0.066	0.589	***<0.001***	0.172	***<0.001***	0.120	*0.014*	1.000	1.000	0.269	1.000	0.067	0.454
Reaction type (clipping)	1,971	0.202	0.033	0.077	−0.004	NA	4.167	NA	***<0.001***	0.657	0.453	***0.002***	***<0.001***	NA	NA	NA	***<0.001***	NA	NA
Reaction location (clipping)	1,971	0.178	0.326	<0.001	−0.012	NA	0.235	NA	***0.008***	*0.018*	1.000	***<0.001***	0.481	NA	NA	NA	0.479	NA	NA
Time lag (clipping)	1,971	0.056	<0.001	0.104	−0.003	NA	0.704	NA	0.257	1.000	0.705	0.160	*0.017*	NA	NA	NA	***0.006***	NA	NA
Second flush type 2014	2,957	0.269	0.480	<0.001	0.007	0.733	0.599	1.303	*0.021*	0.059	1.000	***0.009***	0.249	0.710	1.000	0.365	1.000	1.000	1.000
Second flush type 2016	3,063	0.135	0.415	0.369	0.010	0.576	0.086	−0.303	***<0.001***	***<0.001***	***<0.001***	***<0.001***	*0.013*	0.129	1.000	1.000	0.272	***0.009***	1.000

Note

*Note*. Details as in Table 2.

All *Picea* had a new leader shoot at the end of the experiment (Figure 3b). Some heavily clipped *Picea*(5.2%) reacted with an “unclear” new leader shoot in the first year after clipping, owing to side shoots that were not fully bent upward. “Reaction type” but not “location of reaction” differed between light and heavily clipping treatments (Table 5). *Picea* mostly reacted by forming shoots out of distal buds on the remaining stem piece of the 2014 height increment after light clipping and by flagging a branch in the uppermost shoot whorl after heavy clipping. After light clipping, *Picea*with leader shoots formed out of distal buds were taller than saplings that used flagging, while trees with flagging were taller after heavy clipping than saplings that reacted by forming a new leader out of basal buds (Figure 3b).

Population differences were significant for all growth traits and some quality measures (Tables 4 and 5). Growth and crown quality increased with increasing temperature (*MAT* and *MTsp*), *DTAsp,* and *DDEG*at the seed source (Figure 4b, Table 6). The correlation with continentality was significant for height, height growth, and number of whorl shoots, but not for diameter and biomass. Increasing elevation, precipitation, and *DMI* resulted in less growth and lower quality traits. Of the soil variables, a higher C/N ratio and higher percentage of sand led to more growth and higher quality traits, while the opposite was found for the percentage of clay and available water capacity (Table 6). The number of whorl shoots correlated with sapling height (corr = ca. 0.7) as well as with the type of second flushing in 2016 (corr = 0.77). Thus, both traits had significant population effects and showed correlations with the environmental variables that were in the same direction as correlations between environmental variables and sapling height. For example, with higher temperature at the seed source, more *Picea* had an early prolepsis with a second flush without bud dormancy and then a late prolepsis with a third flush after bud dormancy. However, the 632 saplings with clear elongation growth of their proleptic leader shoot (longer than 2 cm) did not differ among treatments or populations regarding the total length of the proleptic shoot (Table 4).

**Table 6:** Trait–environment relationships between sapling trait provenance effects and environmental variables for 72 provenances of *Picea abies*, displayed using Pearson correlation coefficients

Trait	Geography and topography				Soil properties							Temperature						Precipitation		
	Elevation	Latitude	Longitude	Slope	Sand	Clay	N_tot	C_tot	C_N	pH of top layer	AWC	MAT	MTSp	DTAsp	cont	DDEG5	SFROv	PREC	DRYPsu	DMI
Diameter 2014	***−0.630***	0.072	***−0.292***	−0.127	***−0.345***	***0.225***	***−0.289***	***−0.287***	***−0.225***	***0.192***	***0.197***	***0.730***	***0.729***	***0.529***	0.128	***0.726***	***−0.325***	***−0.498***	***0.397***	***−0.646***
Diameter 2015	***−0.592***	0.053	***−0.256***	−0.097	***−0.289***	***0.209***	***−0.234***	***−0.227***	***−0.218***	0.171	***0.175***	***0.678***	***0.681***	***0.493***	0.111	***0.676***	***−0.296***	***−0.467***	***0.374***	***−0.606***
Diameter 2016	***−0.667***	0.075	***−0.257***	−0.162	***−0.278***	***0.186***	***−0.254***	***−0.236***	***−0.177***	0.164	***0.190***	***0.746***	***0.750***	***0.527***	0.103	***0.746***	***−0.357***	***−0.490***	***0.430***	***−0.644***
DI	***−0.651***	0.082	***−0.213***	***−0.182***	***−0.211***	0.147	***−0.204***	***−0.175***	−0.136	0.136	***0.183***	***0.707***	***0.715***	***0.489***	0.077	***0.712***	***−0.362***	***−0.448***	***0.428***	***−0.597***
Height 2014	***−0.726***	***0.281***	***−0.202***	***−0.265***	***−0.322***	***0.285***	***−0.186***	***−0.211***	***−0.240***	0.088	***0.224***	***0.720***	***0.738***	***0.655***	***0.266***	***0.736***	***−0.314***	***−0.375***	***0.459***	***−0.558***
Height 2015	***−0.686***	***0.407***	−0.088	***−0.310***	***−0.324***	***0.330***	−0.060	−0.117	***−0.236***	0.077	***0.190***	***0.626***	***0.654***	***0.584***	***0.264***	***0.648***	***−0.203***	***−0.213***	***0.417***	***−0.401***
Height 2016	***−0.647***	***0.387***	−0.070	***−0.336***	***−0.348***	***0.326***	−0.031	−0.068	***−0.195***	0.086	***0.200***	***0.582***	***0.611***	***0.583***	***0.284***	***0.605***	***−0.206***	***−0.221***	***0.430***	***−0.394***
Tree height 2014	***−0.729***	***0.297***	***−0.196***	***−0.286***	***−0.314***	***0.276***	***−0.188***	***−0.213***	***−0.230***	0.068	***0.235***	***0.717***	***0.735***	***0.645***	***0.258***	***0.733***	***−0.313***	***−0.365***	***0.472***	***−0.549***
Tree height 2015	***−0.713***	***0.367***	−0.118	***−0.304***	***−0.328***	***0.314***	−0.105	−0.154	***−0.251***	0.101	***0.184***	***0.670***	***0.696***	***0.629***	***0.285***	***0.692***	***−0.218***	***−0.294***	***0.449***	***−0.479***
Tree height 2016	***−0.652***	***0.388***	−0.071	***−0.338***	***−0.349***	***0.325***	−0.031	−0.068	***−0.197***	0.087	***0.200***	***0.587***	***0.616***	***0.587***	***0.286***	***0.611***	***−0.209***	***−0.226***	***0.434***	***−0.399***
Shoot length 2014	***−0.509***	***0.346***	−0.017	***−0.198***	***−0.264***	***0.250***	−0.156	***−0.190***	***−0.246***	0.002	***0.291***	***0.472***	***0.496***	***0.535***	***0.318***	***0.507***	−0.076	***−0.281***	***0.301***	***−0.394***
Shoot length 2015	***−0.594***	***0.427***	−0.038	***−0.318***	***−0.351***	***0.359***	−0.062	−0.138	***−0.242***	0.065	0.152	***0.533***	***0.561***	***0.551***	***0.302***	***0.555***	−0.114	***−0.174***	***0.395***	***−0.343***
Shoot length 2016	***−0.500***	***0.398***	0.017	***−0.391***	***−0.317***	***0.291***	0.017	−0.019	−0.141	0.002	***0.232***	***0.402***	***0.430***	***0.478***	***0.280***	***0.429***	−0.151	−0.107	***0.379***	***−0.238***
Biomass 2014	***−0.670***	0.136	***−0.270***	−0.168	***−0.357***	***0.237***	***−0.284***	***−0.288***	***−0.251***	***0.172***	***0.220***	***0.745***	***0.748***	***0.533***	0.124	***0.748***	***−0.317***	***−0.485***	***0.421***	***−0.637***
Biomass 2016	***−0.692***	0.137	***−0.230***	***−0.202***	***−0.293***	***0.202***	***−0.245***	***−0.233***	***−0.199***	0.150	***0.214***	***0.747***	***0.756***	***0.530***	0.109	***0.755***	***−0.342***	***−0.471***	***0.448***	***−0.628***
Crown form 2016	***0.337***	***−0.286***	0.129	***0.374***	***0.265***	***−0.307***	−0.116	−0.061	***0.213***	***−0.201***	−0.101	***−0.290***	***−0.294***	***−0.265***	−0.118	***−0.279***	0.129	0.018	***−0.274***	0.134
Whorl shoots 2013/2014	***−0.663***	***0.376***	−0.132	***−0.370***	***−0.290***	***0.278***	−0.099	−0.167	***−0.268***	0.163	0.075	***0.611***	***0.636***	***0.565***	***0.230***	***0.623***	***−0.266***	***−0.236***	***0.400***	***−0.412***
Whorl shoots 2015/2016	***−0.673***	***0.410***	−0.136	***−0.334***	***−0.329***	***0.340***	0.012	−0.100	***−0.301***	***0.226***	0.008	***0.589***	***0.618***	***0.506***	***0.194***	***0.606***	***−0.184***	−0.165	***0.383***	***−0.343***
Reaction location (clipping)	0.079	−0.126	−0.021	***0.186***	***0.247***	***−0.257***	***−0.252***	***−0.226***	0.064	***−0.310***	0.138	−0.123	−0.128	***−0.214***	***−0.221***	−0.114	***−0.216***	0.139	0.021	***0.174***
Second flush type 2016	***−0.684***	***0.468***	−0.110	***−0.447***	***−0.334***	***0.324***	−0.013	−0.068	***−0.217***	0.010	***0.260***	***0.590***	***0.616***	***0.515***	***0.181***	***0.618***	***−0.305***	***−0.228***	***0.512***	***−0.376***

Note

*Note*. Details as in Table 3.

The “reaction type,” time lag and multistemming after clipping were not influenced by population differences (Table 5). However, the buds or branches of the new shoots originated lower down the stem with a higher DMI, decreasing continentality (Figure 4b), smaller *DTAsp*, lower soil pH, less clay but more sand in the soil, and steeper slope at the seed source (Table 6).

Frost damage affected 199 (6.5%) of the 3,063 *Picea*still alive in autumn 2016. Of these frost damaged *Picea*, 31.7% had a time lag in their reaction of forming a clear new shoot of one year and eight saplings had a time lag of two years. The cumulative link mixed models did not converge to allow analysis of “reaction type” and “time lag” for this small number of damaged *Picea*(thus not included in Table 5). The model for “frost damage occurrence” did not reveal any significant differences between the provenances or families of *Picea*(*p* = 0.1519).

## 4 DISCUSSION

Knowledge is needed about genetic influences on multiple species to form recommendations in the face of climate change and under the current high ungulate pressure. We analyzed intra‐specific differences of two of the most important tree species in Switzerland (Cioldi et al., 2010), which differ in their selection by wild ungulates—that is, *A. alba* is selected much more often than *P. abies* (Vacek et al., 2014).

### 4.1 How do *A. alba* and *P. abies* saplings react to simulated browsing and are their reactions dependent on population differences?

There were intrinsic differences in the reaction to simulated browsing between the two species. Almost no *Abies*reacted with the bending upward of a previously existing branch, while for *Picea*flagging was very common after light and heavy clipping. Generally, flagging seems to be an efficient but rather rare reaction type for *Abies*saplings (Kupferschmid & Bugmann, 2013), in particular under natural browsing (Kupferschmid, Wasem, & Bugmann, 2015). The different reactions of the species to clipping could be caused by the plagiotropic growth of *Abies*but not *Picea* branches; that is, *Abies*follows the architectural model MASSART and *Picea*follows RAUH (Hallé & Oldeman, 1970). Plagiotropic growth of branches is said to be genetically determined (Bartels, 1993). Based on the results of our study, this genetic effect seems to operate on a species level rather than a within‐species level, as the population effect for “reaction type” was not significant for either species.

The production of a “new basal shoot” out of a bud on the uppermost whorl was the most frequent reaction of *Abies* to heavy simulated browsing and frost damage. Epicormic shoots were also more frequent for *Abies*than for *Picea*. We did not analyze if these basal shoots were formed from small axillary buds at the base of the whorl branches that had remained dormant because of apical dominance or if they were adventitiously formed buds (Meier, Saunders, & Michler, 2012). In any case, these buds were not visible by eye before browsing. Tree vigor may play a major role in the formation of epicormic shoots, with less vigorous trees forming more such shoots, especially after pruning (Meier et al., 2012). *Abies*has a greater potential for forming epicormic shoots because it has more and longer‐living dormant (inactive according to Bonser & Aarssen, 1996) meristems compared with *Picea*(Gruber, 1994). Some evidence was found that differences in epicormic branch production are based on population‐level heritability (Meier et al., 2012). We found population differences for the number of regularly formed buds on the leader shoots formed in 2014 of *Abies* but not for *Picea* and not for the reaction type of either examined species. Thus, further studies are needed to determine whether there are population differences regarding regularly formed buds but not dormant or adventitiously formed buds in *Abies*.

Overall, *Picea* was able to fully compensate height loss induced by a single clipping through the growth of regularly formed distal buds (after light clipping) or by the upward bending of existing branches (heavy clipping). *Abies*, in contrast, partly compensated for height loss only in the second year after leader bud removal or leader shoot loss, that is, saplings had equal shoot length in comparison with not clipped saplings only in the second year after the loss of the apical meristem. However, owing to the considerably faster growth of *Picea*, which resulted in *Picea* saplings that were twice as tall as *Abies*saplings, it is not possible to determine whether the recovery of these two species differed. The very shade tolerant, deep rooting *Abies* were clearly more stressed than *Picea* at our fully sun‐exposed site on shallow soil, and this may have been the reason for the smaller growth and relatively poor recovery of *Abies*.

One of the weaknesses of our experiment was that we had no replication. Our results would be more informative if the experiment had been carried out at two or more locations or even with a fully reciprocal experimental design. However, no interactions between treatment and site were found for morphological traits regarding the recovery of *Fagus sylvatica* saplings when results from our study sites were compared with those at the lowland study site Birmensdorf (Frank et al., 2019). In addition, a reciprocal common garden experiment can be performed with only a few populations and not with seeds from 90 *Abies* and 72 *Picea* seed sources. Another limitation of our experiment is that it was carried out with only a single generation and without knowing the specific genotypes of the populations. We thus interpret our results with caution and recommend selecting specific populations of particular interest for conducting further in‐depth analysis under more natural—that is, at least partly shaded—conditions.

### 4.2 Do different populations recover differently following leader shoot loss?

In our study, the reaction type had an influence on the capacity to recover following browsing (Figure 3). However, reaction type did not depend on population. This suggests no direct involvement of the different seed sources in the recovery via the reaction type. For both species, however, population‐related differences regarding recovery were found, that is, differences in the time lag for *Abies* and in the location of reaction for *Picea*.

One‐third of the *Abies* saplings only formed a new bud and not a real new shoot in the first years after clipping. Thus, the reaction was delayed for one or two years for *Abies*and the saplings were not able to recover the height loss. Such time lags in reaction to browsing for *Abies* have already been detected in many studies (Kupferschmid, Zimmermann, & Bugmann, 2013). There was a significant population effect on the time lag, and it occurred irrespective of treatment intensity. This suggests that the time lag for *Abies*has a heritable basis and is not just due to light or heavy browsing. To our knowledge, this is the first time population‐related differences in the delay in the reaction to clipping, and thus in the recovery, have been shown.

Reactions to clipping were immediate for *Picea* and thus independent of seed source. However, the “location of reaction” differed among *Picea* populations at our site (see below).

### 4.3 Are there correlations between climatic and edaphic conditions at the seed source and the population differences in the recovery following browsing?

In our study, *Abies* populations from lowland locations with a warmer climate at the seed source reacted faster after clipping than highland populations from colder climates (Figure 4a). However, lowland populations were also taller, thicker and had more aboveground biomass. Evergreen conifers have previously been found to retain reserves, particularly in the youngest age class of needles (Millard, 1995). It is therefore likely that the larger lightly clipped *Abies* had more needles available from which reserves for a reaction could be mobilized. Thus, our results support earlier findings that the more vigorously an *Abies* is growing, the faster it reacts after simulated browsing (Kupferschmid & Bugmann, 2013). The vigorous growth of *Abies*additionally seems to be dependent on seed source (Table 1; Hansen & Larsen, 2004; Szeligowski et al., 2011). Albeit, the population differences occurred for lightly and heavily clipped *Abies* and tended to be even larger for heavily clipped saplings that had all their youngest needles cut (Figure 4a). Thus, it is likely that, among others, a population‐dependent mechanism controls the time needed for a reaction and hence the recovery following leader‐shoot loss in *Abies*.

For *Picea*, the “location of reaction” differed among populations, in that trees reacted more efficiently by using the uppermost possible buds or branches when they came from regions with more climate variation over the year (Figure 4b). As “location of reaction” correlated negatively with diameter, height, and aboveground biomass (Pearson correlation around −0.2), it seems that more vigorously growing *Picea*reacted more efficiently. For *Picea*, this vigorous growth is at least partly inherited (Table 4; Frank et al., 2017), as population differences in height and diameter have not been found on all sites (Burger, 1941).

### 4.4 Are the genetic differences in the growth of *A. alba* and *P. abies* saplings maintained in the presence of light and heavy browsing?

First, we were able to confirm that population differences occur for both species in the absence of browsing (e.g. results for the 2014 traits). Second, we tested the hypothesis ungulate browsing does not interfere with growth to such a degree that it counters the growth advantages of the most vigorous populations at our site. We found no evidence that this was the case. In contrast, browsing increased the population differences in height, diameter, and biomass.

For *Abies*, the main reason for this finding is that many slow‐growing *Abies* saplings from colder high‐elevation seed sources needed one or more years to form a new real leader shoot at our site. This corresponds well with the fact that the often‐observed regeneration failure of *Abies* is more accentuated in mountain than lowland forest in Switzerland (e.g. the tolerable browsing limits were derived for mountain forests, cf. Eiberle & Nigg, 1987).

For *Picea* as well, the population differences have increased for all growth traits since the onset of the experiment. The recovery of *Picea* saplings after this single clipping event was good overall, and we would expect major population differences after repeated browsing, owing to the larger influence on the traits (see review of simulated clipping experiments in Kupferschmid, 2017), and after frost damage.

### 4.5 Frost damage versus browsing

Late spring frost can cause equally severe (*Abies*) or even worse (*Picea*) damage to tree saplings than the browsing simulated in our experiment. Populations with poor winter‐frost resistance can have high mortality rates at sites where late frosts occur (Hansen & Larsen, 2004). Our results suggest that populations of *Abies*from warmer and more humid seed sources may be more sensitive to frost than populations with colder and drier conditions at the seed source. Larsen (1986) found particularly large variations in the fast‐growing Calabrian populations of *A. alba*, with increasing frost resistance occurring with increasing elevation. It also seems that high‐elevation populations of *Picea*have greater frost resistance than lowland populations because they have fewer proleptic shoots (Gruber, 1994), but we had too few frost damaged *Picea*to analyze this aspect. Thus, lowland populations are probably overall more prone to leader damage by frost.

## 5 CONCLUSIONS

Based on common garden experiments with *A. alba*, some authors have concluded that there is no need to select for specific populations, as *Abies*is a very adaptable species (Frank et al., 2017; Vitasse, Delzon, Bresson, Michalet, & Kremer, 2009). We found somewhat smaller population differences for *Abies* than for *Picea*saplings, but 17 out of 28 variables still showed important population effects on the *Abies*saplings in our study (Tables 1 and 2). In addition, for three‐ to six‐year‐old saplings, the temporal trend of heritability estimates for total height were found to increase with age in one study (Mihai, Mihaigmihai, & Duta, 2014). However, differences in height after six growing seasons were found to be much larger than after 46 growing seasons in another study (Kerr, Stokes, Peace, & Jinks, 2015). Thus, the population differences in growth and quality traits of *Abies*are probably underestimated and most pronounced in the sapling stage, which coincides with the time of exposure to ungulate browsing.

We found that the existing differences among populations increased because of differences in the capability of saplings to recover growth after a frost event or simulated browsing. Lowland populations from warmer climates grew faster, and for *Picea*also qualitatively better, and recovered faster following leader shoot loss (*Abies*) or reacted with new growth at the uppermost meristem *(Picea*). Thus, even single browsing events can hamper the growth of trees, at least *Abies* saplings, but populations with fast growth can be expected to react rapidly and efficiently to leader shoot damage.

Browsing recovery should be incorporated into forest manager decisions regarding the strategy for regeneration, especially in heavily browsed areas. The interactive effects of site conditions, seed source, and population differences in recovery to stress caused by browsing and frost should be considered carefully. Taking these effects into account could make an important and thus cost‐effective contribution to ensuring that our forests steadily provide their ecosystem goods and services, such as protection from natural hazards, biodiversity preservation, and timber production.

## CONFLICT OF INTEREST

None declared.

## AUTHORS’ CONTRIBUTIONS

AK and CH conceived the ideas, acquired funding, and designed methodology. CH was responsible for the acquisition of data before clipping, while AK collected all data during and after clipping. AK analyzed the data, and both authors interpreted the results. AK led the writing of the manuscript. Both authors contributed critically to the drafts and gave final approval for publication.

## Data Availability

Data from the clipping experiment will be archived at EnviDat with a DOI.
