# Supplementary material for: Recovery of Abies alba and Picea abies saplings to browsing and frost damage depends on seed source
Source: Ecol Evol. 2019 Feb 27;9(6):3335–54. doi: 10.1002/ece3.4955 (PMC6434554; doi:10.1002/ece3.4955)
Supplement: Supplementary file 1 [file ECE3-9-3335-s001.docx]

**Supporting Information**

# Recovery of *Abies alb*a and *Picea abies* saplings to browsing and frost damage depends on seed source

Andrea Doris Kupferschmid & Caroline Heiri

Ecology and Evolution

Figure_1_SuppInfo: Origin of new leader shoot (i.e. reaction type) of *Abies* *alba* (a) and *Picea abies* (b) saplings after clipping in spring 2015 and after frost damage in 2013/2014 for each clipping treatment. Possible ‘reaction types’ were:

- ‘leader shoot’ (control saplings),
- ‘no reaction’ = no new leader shoot formed until the end of the second growing season,
- ‘basal’ = production of a new basal shoot out of a bud on a whorl,
- ‘distal’ = production of a new distal shoot out of a bud on the stem or on the remaining leader shoot pieces,
- ‘flagging’ = upward bending of a previously existing whorl or internodal side shoot,
- ‘epicormic’ = shoot that developed before the stress but was one or more years younger than the regular whorl shoots ([preventitious shoots after Gruber, 1994](#_ENREF_13)),
- ‘side bud’ = in cases of damaged *Picea,* some saplings produced a new leader shoot out of a ‘side bud’ that, without frost damage of the leader bud, would have become a normal whorl shoot.

Table_1_SuppInfo: Description of the continuous growth traits measured on *Abies alba* and *Picea abies* saplings before (2014) and after (2015 and 2016) simulated browsing. Trait = Abbreviation of trait used in the main text, Units = scale of measurement, Year = survey year (measurements took place in late August to October of the respective year). Derived traits are given in italics.

| **Trait** | **Units** | **Year** | **Description** |
| --- | --- | --- | --- |
| Diameter 2012 | mm | 2014 | Sapling stem diameter 2 cm above the ground after growing season 2012, i.e. used as covariate in stat. models. |
| Diameter 2014 | mm | 2014 | Sapling stem diameter 2 cm above the ground after growing season 2014. |
| Diameter 2015 | mm | 2015 | Sapling stem diameter 2 cm above the ground after growing season 2015. |
| Diameter 2016 | mm | 2016 | Sapling stem diameter 2 cm above the ground after growing season 2016. |
| *DI (D2016-D2014)* | *mm* |  | *Diameter increment = Diameter 2016 minus Diameter 2014.* |
| Height 2012 | cm | 2014 | Sapling height measured as vertical distance from the ground surface to the tip of the leader shoot after growing season 2012 = Height of tree top 2012 (equal at that stage); i.e. used as covariate in stat. models. |
| Height 2014 | cm | 2014 | Sapling height measured as vertical distance from the ground surface to the tip of the leader shoot after growing season 2014. |
| Height 2015 | cm | 2015 | Sapling height after growing season 2015. |
| Height 2016 | cm | 2016 | Sapling height after growing season 2016. |
| Tree height 2014 | cm | 2014 | Height at the highest point of the tree, regardless of whether it was a leader shoot or a branch; i.e., height of tree top. |
| Tree height 2015 | cm | 2015 | Height of tree top after growing season 2015. |
| Tree height 2016 | cm | 2016 | Height of tree top after growing season 2016. |
| Shoot length 2014 | cm | 2014 | Length of terminal shoot along the stem axis from growing season 2014. |
| Shoot length 2015 | cm | 2015 | Length of terminal shoot along the stem axis from growing season 2015. |
| Shoot length 2016 | cm | 2016 | Length of terminal shoot along the stem axis from growing season 2016. |
| Second flush length 2016 | cm | 2016 | Length of second and potential additional flushes of *Picea* including all three Second flush types: (1) with bud dormancy, (2) without bud dormancy or (3) combination of type 1 and 2. *Abies* had no second flushes. |
| *Biomass 2014* | *g* | *2014* | *Sapling above-ground ‘dry weight’ after growing season 2014, calculated separately for both species:*  *Abies: ln(dry weight 2014) ≈ -2.5386 + 2.4361*ln(Diameter 2014)*  *Picea: ln(dry weight 2014) ≈ -2.3223 + 2.2002*ln(Diameter 2014) + 0.1791*ln(Height 2014)* |
| *Biomass 2016* | *g* | *2016* | *Sapling above-ground dry weight after growing season 2016, derived from models of the fresh weight 2016 for Abies or from models of diameter and height 2016, derived from the dry weight of 50 Picea from control blocks.*  *Abies: dry weight 2016 ≈ 2.0807 + 0.453719*Fresh weight 2016*  *Picea: ln(dry weight 2016) ≈ -2.3223 + 2.2002*ln(Diameter 2016) + 0.1791*ln(Height 2016)* |
| Fresh weight 2016 | *g* | *2016* | Sapling fresh weight after cutting 2 cm above soil surface (at diameter measurement); only for *Abies.* |

Table_2_SuppInfo: Description and rationale underlining the sampling for the morphological traits assessed for *Abies alba* and *Picea abies* saplings before (2014) and after (2015 and 2016) simulated browsing. Clipping was carried out in spring 2015, and spring frost events (damage) happened in 2013/2014. Trait = Abbreviation of trait used in the main text, Levels = classes of the ordinal factors, Year = survey year (assessment took place in late August to October of the respective year). Derived traits are given in italics.

| **Trait** | **Levels** | **Year** | **Description and rationale for measurement** |
| --- | --- | --- | --- |
| Multi-stemming 2014 | 0,1,2,3 | 2014 | Number of vertically growing stems in 2014 higher than the shoot whorl formed in 2013/2014. For *Abies*, potential leaders had needles around the whole shoot (not like twigs). (3) = 3 or more leader shoots. |
| Multi-stemming 2015 | 0,1,2,3 | 2015 | Number of vertically growing stems in 2015 higher than the uppermost twig whorl. |
| Multi-stemming 2016 | 0,1,2,3 | 2016 | Number of vertically growing stems in 2016 higher than the uppermost twig whorl. |
| Quality 2016 | 1,2,3,4 | *2016* | *Quality in four classes as a combination of Crown form 2016 (C) and Stem form 2016 (S), i.e. C-S: (1) = `1-1`, `1-2`, `2-1`, `2-2`. (2) =`3-1`, `3-2`, `2-3`, `1-3`. (3) =`4-1`, `4-2`. (4) = `5-1`, `5-2`, `5-3`, `4-3`, `3-3`* |
| Crown form 2016 | 1,2,3,4,5 | 2016 | Sapling crown form. Five levels; optimal (1) to low quality (5) |
| Stem form 2016 | 1,2,3 | 2016 | Sapling stem form. 1: straight; 2: bent (deviation from vertical line 22.5–45°); 3: severely bent. |
| Vitality 2016 | 1,2,3,4 | 2016 | Vitality classes: vital (0) to presence of several completely withered twigs (4). |
| Whorl shoots 2013/2014 | 0 to x | 2014 | Twigs in the uppermost whorl in 2014 on the highest leader shoot; count of 0 to 5 for *Abies* and 0 to 13 for *Picea*  Twigs from this whorl could potentially be used to build a new leader shoot by flagging upward, i.e. reaction type 4 from the uppermost whorl (reaction location 1). |
| Whorl shoots 2015/2016 | 0 to x | 2016 | Twigs in the uppermost whorl in 2016 on the highest leader shoot; count of 0 to 6 for *Abies* and 0 to 14 for *Picea* |
| Buds on leader shoot 2014 | 0 to x | 2014 | Number of visible buds on the leader shoot in 2014; count of 0 to 11 for *Abies* and 0 to 16 for *Picea.* Visible buds on the leader shoot were in another clipping treatment with *Abies alba* (Kupferschmid & Bugmann 2013) the prerequisite being a reaction with ‘new distal shoot’ (reaction type 1) |
| Reaction type (clipping) | 1,2,3,4,5,6 | 2016 | Reaction types: (1) production of ‘new distal shoot’ out of a bud on the stem or on the remaining leader shoot pieces, (2) production of ‘new basal shoot’ out of a bud on a whorl, (3) ‘flagging’ of an existing internodal side shoot, (4) ‘flagging’ of an existing whorl shoot, (5) ‘epicormic shoot’, (6) no reaction. For *Picea* there was no (6) because all trees reacted to clipping. |
| Reaction location (clipping) | 1,2,3 | 2016 | Origin of new leader shoot: reaction out of (1) ‘uppermost shoot whorl’, (2) ‘lower shoot whorl’, (3) ‘no reaction’. For *Picea* there was no (3) because all trees reacted to clipping. |
| Time lag (clipping) | 0,1,2 | 2016 | Time lag between clipping and formation of a clear new leader shoot (>0.5 cm). For *Picea* there was no (2) because all trees reacted to clipping. |
| Reaction type (damage) | 1,2,3,4,5,6 | 2015 | Reaction type 3–4 seasons after frost damage, defined in analogy to reaction type after clipping |
| Reaction location (damage) | 1,2,3 | 2015 | Reaction location 3–4 seasons after frost damage, defined in analogy to reaction location after clipping. |
| Time lag (damage) | 0,1,2,3 | 2016 | Time lag between frost damage and formation of a clear new leader shoot (>0.5 cm). (3) = 3 or 4 years |
| Second flush type 2014 | 0,1,2 | 2014 | Type of second (and additional) flushes during growing season 2014. Same as Second flush type 2016 but without (3). |
| Second flush type 2016 | 0,1,2,3 | 2016 | Type of second (and additional) flushes during growing season 2016. (0) no second flush at the 2016 leader shoot, (1) a second flush with bud dormancy, (2) a second flush without bud dormancy, or (3) a second flush with a combination of dormancy and no dormancy. |

Kupferschmid A.D. & Bugmann H. (2013). Timing, light availability and vigor determine the response of *Abies alba* saplings to leader shoot browsing. European Journal of Forest Research. 132:47–60.
